# Allometric Scaling Reveals Evolutionary Constraint on Odonata Wing Cellularity via Critical Crack Length

**DOI:** 10.1002/advs.202400844

**Published:** 2024-04-13

**Authors:** Shahab Eshghi, Hamed Rajabi, Shaghayegh Shafaghi, Fatemeh Nabati, Sana Nazerian, Abolfazl Darvizeh, Stanislav N. Gorb

**Affiliations:** ^1^ Department of Functional Morphology and Biomechanics Zoological Institute Kiel University 24118 Kiel Germany; ^2^ Division of Mechanical Engineering and Design School of Engineering London South Bank University London SE1 0AA UK; ^3^ Mechanical Intelligence Research Group South Bank Applied BioEngineering Research (SABER) School of Engineering London South Bank University London SE1 0AA UK; ^4^ Department of Mechanical Engineering Ahrar Institute of Technology and Higher Education Rasht 4193163591 Iran; ^5^ Department Artificial Intelligence in Biomedical Engineering Friedrich‐Alexander‐Universität Erlangen‐Nürnberg Henkestraße 91 91052 Erlangen Germany; ^6^ Faculty of Mechanical Engineering University of Guilan Rasht 4199613776 Iran

**Keywords:** computer‐vision, entomology, image‐processing, MATLAB, morphology

## Abstract

Scaling in insect wings is a complex phenomenon that seems pivotal in maintaining wing functionality. In this study, the relationship between wing size and the size, location, and shape of wing cells in dragonflies and damselflies (Odonata) is investigated, aiming to address the question of how these factors are interconnected. To this end, WingGram, the recently developed computer‐vision‐based software, is used to extract the geometric features of wing cells of 389 dragonflies and damselfly wings from 197 species and 16 families. It has been found that the cell length of the wings does not depend on the wing size. Despite the wide variation in wing length (8.42 to 56.5 mm) and cell length (0.1 to 8.5 mm), over 80% of the cells had a length ranging from 0.5 to 1.5 mm, which was previously identified as the critical crack length of the membrane of locust wings. An isometric scaling of cells is also observed with maximum size in each wing, which increased as the size increased. Smaller cells tended to be more circular than larger cells. The results have implications for bio‐mimetics, inspiring new materials and designs for artificial wings with potential applications in aerospace engineering and robotics.

## Graphical abstract

This study utilizes WingSegment, a Computer‐Vision tool, to analyze wing and cell size relationships in 389 Odonata wings. Despite varying wing sizes, statistical analysis shows over 80% of cells measure between 0.5 and 1.5 mm, implying a link to the crack‐stopping function in insect wings. Furthermore, an inverse correlation between cell size and circularity suggests smaller cells are more circular.

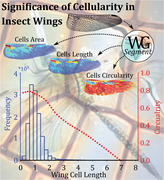

## 1 Introduction

Insect wings are lightweight segmented structures that balance flexibility and stiffness, achieved through an intricate network of veins interacting with other wing components. This balance is required to generate enough lift for insect flight and keep its structural durability even during collisions.^[^ ^1^, ^2^, ^3^, ^4^, ^5^, ^6^ ^]^ One of the fascinating aspects of insect wings is their scaling characteristics, which can vary depending on the species, size, shape, and ecology.^[^ ^7^, ^8^ ^]^ Scaling in insect wings can refer to the relationship between wing size and other parts of the insect body or between the wing size and the size of membranes stretching between veins.^[^ ^9^ ^]^ The veins of an insect's wings form a complex network of interconnected structures that provide support and rigidity to the wing membrane. The interplay between these two components is complex and adaptable, allowing insects to fly and navigate their environments with remarkable efficiency and agility. However, the relationship between wing size and membrane size is still unknown.^[^ ^6^, ^10^, ^11^ ^]^

The wings of the representatives of the order Odonata, including dragonflies and damselflies, have evolved to meet the demands of their flight and maneuverability.^[^ ^12^, ^13^ ^]^ The wings of these insects are composed of a complex array of cells, veins, and membranes arranged in a hierarchical structure. The structural characteristics of insect wings are influenced by the size and shape of the wing cells, as well as the overall size of the wing.^[^ ^14^, ^15^, ^16^, ^17^, ^18^ ^]^ Understanding the scaling relationships between these parameters can provide insights into the mechanics of the wing and potentially inspire the development of bio‐inspired materials and systems.^[^ ^19^, ^20^, ^21^, ^22^, ^23^ ^]^

Previous studies have suggested that different Odonata species show different wing length allometries, meaning that the wing size changes at a different rate than the overall body size.^[^ ^24^, ^25^, ^26^ ^]^ Other studies also suggest that the size of cells in the locust wing relates to the critical crack length, and the cellular structure of the insect wing is a crack resistance strategy to increase durability.^[^ ^14^, ^15^, ^27^ ^]^ The critical crack length denotes the dimension of a crack in a material where the material undergoes catastrophic failure or fracture. In simpler terms, it's the point where the combination of stress and crack size becomes so important that the crack quickly spreads and the material fails. However, whether the scaling in wing cells is allometric or isometric remains unresolved. In this study, we address this question by examining the relationship between wing cell geometry relative to the wing by analyzing 389 wings from 197 species of Odonata using WingGram and WingSegment‐ our previously developed software packages for automated analysis of insect wings.^[^ ^28^, ^29^ ^]^ These tools are designed based on computer vision methodologies, such as region‐growing,^[^ ^30^, ^31^ ^]^ and line simplification^[^ ^32^ ^]^ for extracting geometric features of insect wing images. The wings were selected from seven families of damselflies, including Coenagrionidae, Lestidae, Megapodagrionidae, Perilestidae, Platystictidae, Protoneuridae, and Synlestidae, and nine families of dragonflies, including Aeshnidae, Austropetaliidae, Corduligasteridae, Corduliidae, Gomphidae, Libellulidae, Macromiidae, Neopetaliidae, and Petaluridae.^[^ ^33^ ^]^

## 2 Experimental Section

All 389 wing images used in the study were obtained from the publication by Hoffmann et al.^[^ ^33^ ^]^ All images were edited to ensure no noise would influence the measurements. A MATLAB toolbox called WingSegment^[^ ^34^ ^]^ was used, an improved version of the previously developed toolbox, WingGram.^[^ ^28^ ^]^ WingSegment employed computer vision and mathematical techniques, including region growing,^[^ ^30^, ^31^ ^]^ thinning,^[^ ^35^ ^]^ and line simplification^[^ ^32^ ^]^ algorithms, to extract the geometric characteristics of cells.^[^ ^28^, ^29^, ^36^ ^]^ It required an image of the wing as the input.

Region growing was a method used to identify a domain surrounded by black pixels. This approach considered a randomly selected white pixel within the domain as the initial seed. The process began by examining the neighboring pixels of this seed. If neighboring pixels were white, their color was changed to grey and treated as new initial pixels. Subsequently, in the next iteration, these newly identified white pixels examine their neighbors to identify additional white or black pixels. If a pixel was black, it was considered the domain boundary. This recursive process continues until all white pixels within the domain were examined. Consequently, all black pixels located on the domain of that region were detected. This process was applied to all cells of the wing automatically until all cells were identified. Once the boundary of each region was detected, the maximum distance between two points inside the region was considered the length of that cell. The number of detected white pixels within the region represents the area of the region. To calculate circularity, Equation (1) is employed:

(1) $$C=\left(4\pi *area\right)/\left(perimeter^{2}\right)$$

where “area” represents the area of the domain, and “perimeter” denotes the perimeter of the domain, measured from the information extracted from the wing images using the region‐growing method. Furthermore, the distance between each domain centroid and the wing outer line was measured as the distance between cells and margins. Codes S1, S2, and S3 provided in the Supporting Information correspond to the embedded MATLAB scripts within WingSegment. These scripts facilitate the importation of the wing image, region‐growing, and extraction of the wing cell geometry. Additionally, Code S4 demonstrates the integration of Codes S1‐S3 for extracting wing cell area, length, and circularity. This code also used MATLAB to generate heatmaps depicting the distribution of wing cell area, length, and circularity.

**Figure 1:**
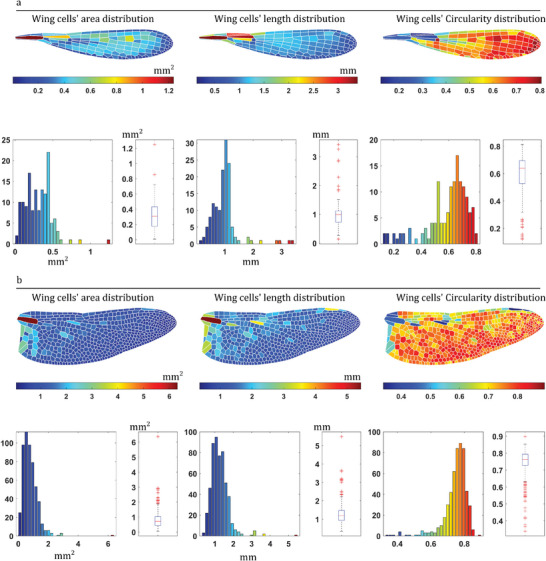
Using WingSegment for extracting the geometric features of wing cells in (a) right hind wing of *Acanthagrion chararum* (Coenagrionidae) with 18.8 mm wing length, and (b) right hind wing of *Aeshna juncea* (Aeshnidae) with 40 mm of wing length. For each wing, the contour distribution, histogram, and box plot representing wing cells' area, length, and width are depicted.

All information on the wings examined in this study, including the distribution contour and histogram of wing cell area, length, and circularity, as well as the Excel files containing the measured values, was documented in the Supporting Information. **Figure** **1** shows an example of what WingSegment generated from the image of *Acanthagrion chararum* (Coenagrionidae) (Figure 1a) and *Aeshna juncea* (Aeshnidae) (Figure 1b).

## 3 Results

In this study, we measured the area, length, and circularity of wing cells from both dragonflies and damselflies, which were selected from various families. We also measured the area of the wings and the distance of cells from wing margins. All data is documented and is available via the Zenodo repository (See Supporting Information), including the family, genus, species, wing position, and length of wings, as well as wing images and the contour of cells' length, area, and circularity.

**Figure 2:**
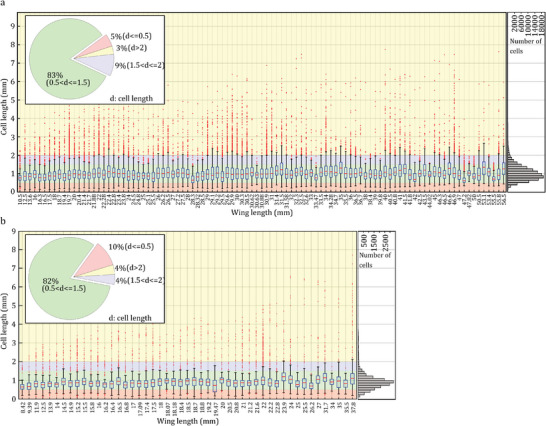
Distribution of cell length in dragonflies (a) and damselflies (b) categorized by wing length. Box plots showing the median, interquartile range, and outliers of cell lengths for each wing size category. Pie chart depicting the proportion of cells falling within different length ranges. Histogram displaying the frequency distribution of cell length ranging from 0.1 to 9 mm.

**Figure 3:**
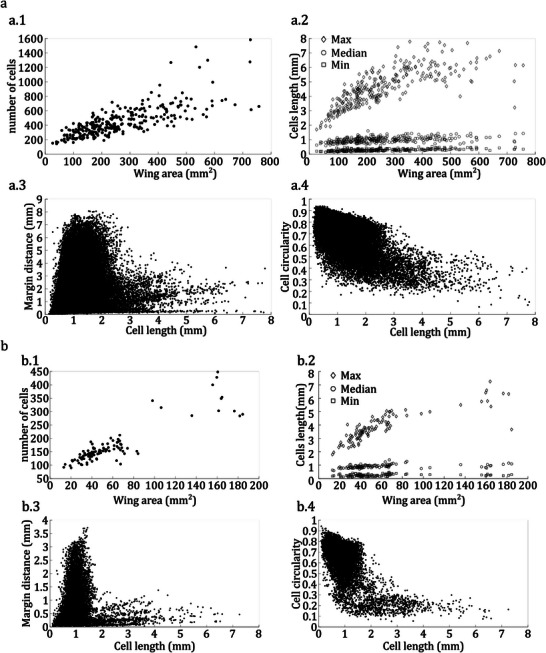
Statistical data on the size and geometry of insect wings for dragonflies (a) and damselflies (b). Each panel contains four scatter plots depicting 1) the relationship between the number of cells and the area of the wing, 2) changes in the maximum, minimum, and median of cell length with wing area, 3) changes in the cell length based on the distance from the wing margins, and 4) changes in the cell circularity with cell length.

**Figure** **2** shows the cell length distribution based on the wing length for dragonflies (a) and damselflies (b). Although the wing length varied widely from 10.5 to 56.5 mm in dragonflies and 8.42 to 37.80 mm in damselflies, the cell length in both dragonflies and damselflies varied only slightly, ranging from about 0.1 to 8mm. However, we found that most cells have a length between 0.5 and 1.5 mm, with only a small percentage being less than 0.5 mm or more than 1.5 mm. Specifically, 83% and 82% of cells had a 0.5–1.5 mm length in dragonflies and damselflies, respectively. The additional information presented in **Figure** **3** provides valuable insights into the shape and position of cells in both dragonflies and damselflies. Moreover, **Table** **1** represents the results of the statistical analysis between the wing, and its cells in dragonflies and damselflies.

**Table 1:** Correlation analysis between wing characteristics and cell properties in dragonflies and damselflies.

Variable Pair		Dragonfly		Damselfly	
		Correlation	P‐Value	Correlation	P‐Value
Wing Area	max(Cell Length)	0.76	<<0.001	0.84	<<0.001
Wing Area	median(Cell Length)	0.46	<<0.001	0.37	<<0.001
Wing Area	min(Cell Length)	0.56	<<0.001	0.18	0.06
Wing Area	Number of Cells	0.77	<<0.001	0.89	<<0.001
Cell Length	Cell Circularity	−0.58	<<0.001	−0.64	<<0.001

## 4 Discussion

Our analysis indicates that the number of cells increases as the wing area increases (Figure 3a.1, b.1). The correlation of 0.77 and 0.89 between the wing area and the number of cells in dragonflies and damselflies supports this conclusion (Table 1). Interestingly, as shown in Figure 3, we observed that regardless of the area of the wings, the minimum cell length is similar across all wings. Besides, the correlation between the wing area and the minimum size of cell length, as stored in Table 1 in all wings in dragonflies and damselflies, is 0.56 and 0.18, respectively. This suggests, especially in damselflies, that there is almost no correlation between the minimum size of the cells and the size of the wing. However, it seems the results for comparing the minimum cell size and the wing size in damselflies are not statistically significant due to having a P‐value of 0.06. Table 1 shows a correlation of 0.76 and 0.84 between the wing area and the maximum cell length in the wings of dragonflies and damselflies, respectively. This shows that the length of the largest cell increases proportionally with wing size (Figure 3a.2, b.2). Notably, our findings reveal that larger cells tend to be located near the margins of both dragonfly and damselfly wings, as depicted in panels a.3 and b.3 of Figure 3. Moreover, we observed that the biggest cells are usually situated in the basal part of the wings. Besides, Figure 3a.3 shows in dragonflies almost more than 99% of cells are located at a distance of more than 3 mm from margins having a length of less than 2.5 mm. This value in damselflies (Figure 3b.3) is 98% for cells with a length less than 1.5 mm and with a distance more than 1mm from the wing margin. Further examination of panels a.4 and b.4 in Figure 3 reveals that smaller cells tend to be more circular than larger cells, which exhibit more significant variation in small cells' shape. A correlation of ‐0.64 between the cell length and the circularity in damselflies and a correlation of ‐0.58 in dragonflies shows a reverse trend between the cell length and circularity. However, the relationship has moderate strength.

The cellular structure of insect wings is highly complex and heterogeneous. Insect wings consist of a variety of cell types, each with its unique structure and function. In addition, the size and shape of these cells can vary widely, depending on their location within the wing and their role in maintaining structural integrity.^[^ ^6^, ^37^ ^]^ One of the most interesting findings of this study is that most of the cells have a length between 0.5 and 1.5 mm, corresponding to the critical crack length of locust wings' membrane.^[^ ^15^, ^38^ ^]^ It is noteworthy that microscopic observations reveal irreparable small cracks in various parts of the wing.^[^ ^2^, ^39^ ^]^ The cellular structure of the wing appears to be a strategy aimed at delaying wing failure. Furthermore, our discoveries indicate that the wing cell length could be optimized to reduce the possibility of cracks and other types of mechanical damage. Nevertheless, it has been previously established that cross veins have an adverse effect on the wing's natural frequency.^[^ ^40^ ^]^

In our previous investigation, we gathered 119 wings from over 30 individual *Sympetrum vulgatum* dragonflies to identify which sections of the wing experience more damage.^[^ ^41^ ^]^ All samples were collected during the latter half of their lifespan while in flight. The results indicate that the probability of damage occurring in the trailing edge and wing tip is higher compared to other regions, likely due to their increased exposure to obstacles during flight. Our findings reveal that the cells in these specific areas exhibit smaller lengths. As a result, we can infer that the cellular structure of the wing could potentially endure wing cracks that occur at the trailing edge or tip, providing a level of resilience in these vulnerable regions. Furthermore, our findings suggest that the basal regions of the wing contain larger cells, which are known to experience stronger aerodynamic forces.^[^ ^42^ ^]^ Consequently, one would expect these areas to exhibit more damage. Surprisingly, our observations of damaged wings^[^ ^41^ ^]^ contradict this expectation. However, it is important to note that we cannot definitively conclude that damage never occurs in the basal areas.^[^ ^43^ ^]^ It is worth considering that the presence of thicker veins and corrugations may contribute to enhancing the resilience of these basal parts.

Moreover, the circularity of cells is crucial in maintaining the cell size in all directions, resulting in the maximum area while using less material.^[^ ^37^ ^]^ In areas of the wing apart from the basal part and leading edge, the thickness of the veins is reduced, a crucial factor in balancing flexibility, stiffness, and lift generation. Although thinner veins may risk increased damage, having smaller‐sized cells enhances the resilience of these wing sections. In such instances, the membrane area is compromised if the cells lack a more circular shape. More cells are needed to compensate and maintain sufficient area, resulting in increased weight. As a result, we assume this is why smaller cells have more circular shapes. It is important to mention that we must consider other wing components while striding the size of cells. The cuticle thickness is non‐uniformly distributed among the wing.^[^ ^44^ ^]^ Also, the wing itself is not a planar structure, but it is entirely corrugated, and the corrugations strongly influence the structural stiffness of the wing.^[^ ^45^, ^46^ ^]^ The relationship between the spatial shape of the wing, the cell size, and the cuticle thickness remains elusive and yet to be understood.

The wing functionality is also affected by its microstructure, which is another crucial factor. Prior research has shown that the wing contains two distinct types of micro joints: fused and flexible.^[^ ^42^, ^47^ ^]^ Fused micro joints are characterized by veins that intersect and come into significant contact with each other, while flexible micro joints have minimal direct contact between veins and are typically connected by a section of resilin‐rich cuticle.^[^ ^48^, ^49^ ^]^ It is also known that the presence of flexible micro joints containing resilin in insect wings can decrease the likelihood of material failure by avoiding stress concentrations at intervals between veins.^[^ ^50^ ^]^ In addition, flexible microjoints are seldom observed in regions with smaller cells,^[^ ^37^ ^]^ suggesting that cell size may be a compensatory mechanism for the absence of these joints.

**Figure 4:**
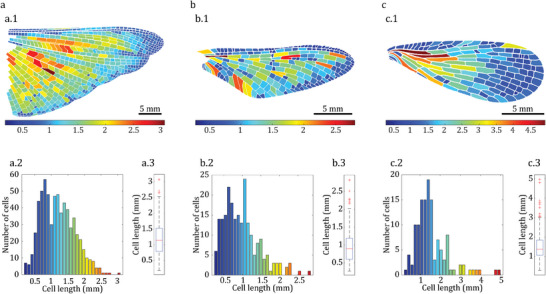
Cell length distribution in three different insects from three different orders. Subpanels (1), (2), and (3) in each panel show the contour plot, histogram, and box plot of cell length distribution, respectively. Panel (a) represents Caelifera (dessert locust), panel (b) represents Ephemeroptera (mayfly), and panel (c) represents Mecoptera (scorpionfly).

The current study suggests that the size of cells on the Odonata wing might be a way to prevent cracks from propagating, but further research can reveal if this is valid for other types of insects. However, significantly larger cellular structures are common in many insects. To explore this further, the wings of a desert locust (**Figure** **4**a), mayfly (Figure 4b), and scorpionfly (Figure 4c) were examined. The results indicate that the trend observed in the Odonata wing is also present in these insects, with many cells having a length between 0.5–1.5mm. This finding provides additional evidence that the cellular structure is a strategy used by many insects, not only Odonata, to maintain the functionality of their wings and extend their lifespan. Ultimately, it is essential to note that insect wings are crafted not just to withstand damage but also to generate lift.^[^ ^51^ ^]^ Various elements interact simultaneously to maintain their functionality, such as micro joints, composite materials, vein thickness, corrugation, and various vein junctions.^[^ ^19^, ^44^, ^49^, ^52^, ^53^ ^]^ This research specifically focuses on the wing's morphometry, particularly the cell sizes, to explore their role in enhancing wing durability. We assert that further in‐depth studies are necessary to uncover the multi‐functionality of insect wings. The data presented in this study is valuable and can contribute significantly to the exploration of insect wings through the application of multi‐objective optimization, machine learning, and deep learning methods.

## 5 Conclusion

In this study, we found that regions with smaller cells tend to lack flexible joints and instead rely on cell size as a compensatory mechanism to maintain wing integrity. In contrast, regions with larger cells often feature flexible joints that prevent stress concentrations and reduce the risk of material failure. Interestingly, we also observed that the length of cells located in the farthest regions from the wing margins is in the critical crack length range. This suggests that there may be a functional trade‐off between cell size and wing size, with smaller cells providing more excellent resistance to crack propagation at the expense of higher material costs and higher local weight. To sum up, examining the cellular makeup of insect wings offers valuable insights into how nature has evolved to optimize strength, flexibility, and damage resistance. By creating biomimetic designs based on studying these structures, new materials and systems that possess unique properties and can be utilized in various fields can be produced. Our research has potential implications for the design of synthetic wings, crack‐resistant meta‐materials, and thin films.^[^ ^54^ ^]^ By comprehending how cells are distributed according to their specific characteristics in insect wings, we can devise better methods to imitate their structural features and improve the longevity of engineering systems for a broad range of applications.

## Conflict of Interest

The authors declare no conflict of interest.

## Author Contributions

S.S., F.N., and S.N. contributed equally to this work. Conceptualization is performed by S.G., S.E., and H.R.; Data Curation is performed by S.E., S.S., F.N., and S.N.; Funding Acquisition is provided by S.E., S.G., A.D., and H.R.; Methodology is performed by S.E., and H.R.; Project Administration is performed by S.G., and H.R.; Resources are provided by S.G., and A.D.; Software is provided by S.E.; Supervision is performed by A.D., S.G., and H.R.; Validation is done by S.E.; Visualization is performed by S.E.; Writing‐ Original Draft Preparation is completed by S.E.; Writing‐ Review & Editing is performed by S.E., F.N., S.S., S.N., S.G., and H.R.

## Supporting information

Supporting Information

## Data Availability

The data that support the findings of this study are openly available in Zenodo at https://doi.org/10.5281/zenodo.10557201, reference number [55].
