# Supplementary material for: Allometric Scaling Reveals Evolutionary Constraint on Odonata Wing Cellularity via Critical Crack Length
Source: Adv Sci (Weinh). 2024 Apr 13;11(23):2400844. doi: 10.1002/advs.202400844 (PMC11187826; doi:10.1002/advs.202400844)
Supplement: Supplementary file 1 — Supporting Information [file ADVS-11-2400844-s001.pdf]

ADVANCED SCIENCE
Open Access

Supporting Information

for *Adv. Sci.*, DOI 10.1002/advs.202400844

Allometric Scaling Reveals Evolutionary Constraint on Odonata Wing Cellularity via Critical Crack Length

Shahab Eshghi, Hamed Rajabi, Shaghayegh Shafaghi, Fatemeh Nabati, Sana Nazerian, Abolfazl Darvizeh and Stanislav N. Gorb*

Supplementary Information for
”Allometric scaling reveals evolutionary constraint on Odonata wing cellularity via critical crack length”

Shahab Eshghi*[1], Hamed Rajabi[2,3], Shaghayegh Shafaghi[4,§], Fatemeh Nabati[4,§], Sana Nazerian[5,§], Abolfazl Darvizeh[†,4,6], and Stanislav N. Gorb[1]

[1]*Functional Morphology and Biomechanics, Institute of Zoology, Kiel University, Kiel, 24118, Germany*
[2]*Division of Mechanical Engineering and Design, School of Engineering, London South Bank 6 University, London, UK 7*
[3]*Mechanical Intelligence Research Group, South Bank Applied BioEngineering Research 8 (SABER), School of Engineering, London South Bank University, London, UK*
[4]*Department of Mechanical Engineering, Ahrar Institute of Technology and Higher Education, Rasht, Iran*
[5]*Department Artificial Intelligence in Biomedical Engineering, Friedrich-Alexander-Universität Erlangen-Nürnberg, Henkestraße 91, 91052, Erlangen, Germany*
[6]*Faculty of Mechanical Engineering, University of Guilan, Rasht, Iran*

§ These authors contributed equally to this work.

Contents

1 Codes **2**
- 1.1 Code S1: Get Image . . . 2
- 1.2 Code S2: Region Growing . . . 2
- 1.3 Code S3: Wing Cell Segmentation . . . 4
- 1.4 Code S4: Wing Geometry Extraction . . . 6

2 Zenodo Repository **8**

3 Documented Files **8**

4 WingSegment and WingGram **9**

*Corresponding author: eshghi.shahab@gmail.com
†The author is deceased. Abolfazl Darvizeh (1951-2021), a great mentor and colleague, passed away before preparing the manuscript.

1 Codes

1.1 Code S1: Get Image

Code S1 is the function developed for importing the wing image. Line 1 in Code S1 defines the function.

Input: The input for this function is not explicitly defined. To import the wing image, we utilized the *uigetfile* function in MATLAB, allowing the user to import a desired file (in this case, the wing image). The output of *uigetfile* is the 'file' name and the 'path' where the file is stored.
Ouput: The outputs of this function is as follows:

- *imageBinaryImage*: This variable stores the binarized image of the wing.
- *imageOrgImage*: This variable stores the original matrix of the imported wing image.
- *imageWidth*: This variable stores the width of the imported image.
- *imageHeight*: This variable stores the height of the imported image.

Code Description: This code reads the image matrix in line 5. Line 6 checks whether the image is in RGB format. If the image is in RGB, the *rgb2gray* function in line 7 converts the image to grayscale. Line 9 is included to binarize the image using the *imbinarize* function. This function is a built-in MATLAB function that utilizes Otsu's method. The function requires a threshold value, and we have set it to 0.54 in this code. Lines 10 to 14 handle the cropping of the image to fit within a frame.

Code S1: GetImage code in MATLAB, for manual importing of the wing Image

```
1  function [imageBinaryImage,imageOrgImage,imageWidth,imageHeight]=GetImage
2  [file,path,indx] = uigetfile( '*.*');
3  ThresholdEditField = 0.54;
4  if indx
5      imageOrgImage = imread([path file]);
6      if size(imageOrgImage,3)==3
7          imageOrgImage = rgb2gray(imageOrgImage);
8      end
9      imageBinaryImage= imbinarize(imageOrgImage,ThresholdEditField); %Otsu's Method
10     [r,c] = find(imageBinaryImage==0);
11     imageBinaryImage = imageBinaryImage(min(min(r))-3:max(max(r))+3,min(min(c))-3:
           max(max(c))+3);
12    imageOrgImage = imageOrgImage(min(min(r))-3:max(max(r))+3,min(min(c))-3:max(max(
          c))+3);
13     imageWidth = size(imageOrgImage,2);
14     imageHeight = size(imageOrgImage,1);
15 end
16 end
```

1.2 Code S2: Region Growing

Region growing, as introduced in the main manuscript, extracts the boundary of a closed domain surrounded by black pixels.
Inputs: Three inputs are defined for Code S2:

- *imageBinaryImage*: The binary image, the first output of Code S1.
- *r*: The row number (y-coordinate) of a random seed inside a desired region.
- *c*: The column number (x-coordinate) of a random seed inside a desired region.

Outputs: Code S2 has four outputs:

- *cell_whiteID*: An N-by-2 array storing the coordinates of detected white pixels inside the desired region, used to generate the corresponding matrix for area, length, and circularity distribution of wing cells.
- *n_white*: The number of detected white pixels in the desired region, representing the area of the detected region.

- *im*: The image after detection of the region. When a desired region in the wing is detected, its color changes to gray to avoid repeated detection.
- *boundary*: An N-by-2 array containing the coordinates of the detected region.

Code Description:

- **line 3:** The binary image's matrix is converted to double because, upon importing an image into the function, the color of the detected area changes to gray, which is not possible in a binary image.
- **Line 4:** *FWP* represents 'Found White Pixels'. Initially, r and c (the second and third inputs of Code S2) go into *FWP*. This variable updates during the algorithm's execution.
- **Lines 5-6:** *border* and *cell_whiteID* are empty matrices that store the coordinates of detected boundary pixels and white pixels inside the region, respectively.
- **Lines 7-34:** The while loop starts from the initial seed point (r, c) and checks for new white (lines 8-18) and black pixels (lines 19-30) around the seed pixel. Each time it detects a new white or black pixel, it saves their coordinates separately. Also, each time it finds white pixels, it changes the color of those detected pixels and continues checking for new white pixels around those detected points. The while loop continues until there are still white pixels inside the region.
- **Lines 35-59:** In the while loop, detected pixels on the boundary are not in order. It means that the coordinates don't define a closed loop, and they are only points on the boundary that are stored irregularly. In lines 35-59, the code constructs a closed loop of those extracted points on the boundary of the domain.

To demonstrate how to run this function and use it for another project, we have developed Code S??. In this code, the user can simply import a desired image and click inside one region, and the code extracts that region's boundary.

Code S2: Region Growing Code in MATLAB

```
1  function [cell_whiteID,n_white,im,boundary]= RegionGrowing(imageBinaryImage,r,c)
2  %r, and c represent the row and column number of a random seed inside the desired
      region
3  im = double(imageBinaryImage);
4  FWP=[c,r];       % FWP:found white pixel(first white pixel is choosen by user)
5  border=[];
6  cell_whiteID=[];
7  while ~isempty(FWP) %the code continues while it does not find new white pixe
8      X=[FWP(:,1) FWP(:,1)     FWP(:,1)+1     FWP(:,1)     FWP(:,1)-1];
9      Y=[FWP(:,2) FWP(:,2)-1     FWP(:,2)     FWP(:,2)+1     FWP(:,2)];
10     X=X(:); Y=Y(:);
11     l=length(X);
12     new_FWP=[];
13     for i=1:l
14         if im(Y(i),X(i))==1
15             im(Y(i),X(i))=0.8; % changes the color of found white pixel to bright
                  grey
16             new_FWP=[new_FWP; X(i) Y(i)];   %#ok %new_FWP: new Found White Pixel
17         end
18     end
19     % Finding new black pixels in the neighbor of FWP on 8 directions
20     X=[FWP(:,1) FWP(:,1)+1 FWP(:,1) FWP(:,1)-1 FWP(:,1)-1 FWP(:,1)-1 FWP(:,1)+1 FWP
          (:,1)+1];
21     Y=[FWP(:,2)-1 FWP(:,2) FWP(:,2)+1 FWP(:,2) FWP(:,2)-1 FWP(:,2)+1 FWP(:,2)-1 FWP
          (:,2)+1];
22     X=X(:); Y=Y(:);
23     l=length(X);
24     new_FBP=[];
25     for i=1:l
26         if im(Y(i),X(i))==0
27             im(Y(i),X(i))=0.1; % changes the color of found black pixel to dark
                  grey
```

```
            new_FBP=[new_FBP; X(i) Y(i)];     %#ok %new_FBP: new Found Black Pixel
        end
    end
    border=[border;new_FBP];                              %#ok
    cell_whiteID=[cell_whiteID;new_FWP];     %#ok
    FWP=new_FWP;
end
border = unique(border,'rows');
n_white=size(unique(cell_whiteID,'rows'),1);
im_bw = zeros(size(im,1),size(im,2));
for i=1:size(border,1)
    im_bw(border(i,2),border(i,1))=1;
    im(border(i,2),border(i,1))=0;
end
BW = imbinarize(im_bw);
try
    boundary = bwtraceboundary(BW , [border(i,2),border(i,1)],'S');
catch
    try
        boundary = bwtraceboundary(BW , [border(i,2),border(i,1)],'E');
    catch
        try
            boundary = bwtraceboundary(BW , [border(i,2),border(i,1)],'N');
        catch
            boundary = bwtraceboundary(BW , [border(i,2),border(i,1)],'W');
        end
    end
end
boundary = [boundary(:,2) boundary(:,1)];
boundary(:,2)=size(im,1)-boundary(:,2);
[boundary(:,1), boundary(:,2)] = poly2cw(boundary(:,1), boundary(:,2));
im = double(im);
end
```

1.3 Code S3: Wing Cell Segmentation

This code is developed to segment all cells of a wing by repeatedly applying the *Region_Growing* function.
Input: The only input of this function is the binary image generated by the *GetImage* function.
Outputs: Code S3 has six outputs as follows:

- *ContourMatrix_Area*: Stores the matrix generated by *RegionGrowing* that contains the corresponding matrix for the distribution of wing cells' areas.
- *ContourMatrix_Length*: Similar to the previous one, this variable contains the distribution of wing cells' lengths.
- *ContourMatrix_Circularity*: This variable similarly contains the distribution of wing cells' circularities.
- *wingOutline*: This is an N-by-2 array that contains the coordinates of the wing outline, extracted from *RegionGrowing*.
- *wingCells*: This is a one-by-N cell array. The number of cells in this array represents the number of wing cells, and each cell array contains the coordinates of one wing cell boundary.
- *wingInfo*: This is an N-by-6 matrix. N represents the number of cells. Each row of this variable has six columns representing the cell index, the x-coordinate of the cell centroid, the y-coordinate of the cell centroid, the cell's area, the cell's length, and the cell's circularity.

Code Description:

- **Lines 8-11:** These lines generate a gray frame around the image. This is necessary for extracting the outline of the wing. Otherwise, the *RegionGrowing* function faces problems when it reaches the edge of the image (first and last columns and rows of the image matrix).

- **Line 16:** *RegionGrowing* runs in this line to extract the outline of the wing.
- **Lines 32-63:** In line 32, a *while* loop starts. In this while loop, the *RegionGrowing* function runs recursively until all cells are detected. As mentioned, the second and third inputs of the *RegionGrowing* function are one seed point inside the region. Keep in mind that the outline is already detected, and as a result, the color of the outer region of the wing image is already gray. To find the first seed inside the first cell, in line 30 the code finds all white pixels, and in line 33, it uses only the first detected white pixel. As the outer region of the wing is gray, this white pixel is located inside one of the cells. Then, *RegionGrowing* extracts that specific cell's boundary and changes the color of that cell to gray. Then inside the loop in line 60, the code again searches for new white pixels, and absolutely these white pixels are inside not detected cells because all detected cells have changed color to gray. This while loop continues until the code finds no new white pixels. Video S3 in the supplementary materials visually demonstrates this procedure.

Code S3: Wing Cells Segmentation Code in MATLAB

```
1  function [ContourMatrix_Area,...
2      ContourMatrix_Length,...
3      ContourMatrix_Circularity,...
4      wingOutline,...
5      wingCells,...
6      wingInfo] = cellSegment(imageBinaryImage)
7  w = double(imageBinaryImage);
8  w(1,:)=.5;
9  w(end,:)=.5;
10 w(:,1)=.5;
11 w(:,end)=0.5;
12 s_im=size(w);
13 r=2; c=2;
14 wingInfo = [];
15 wingCells ={};
16 [cell_whiteID,~,w,boundary]=RegionGrowing(w,c,r);
17 wingOutline= boundary;
18 ContourMatrix_Area=zeros(s_im(1),s_im(2));
19 for i=1:size(cell_whiteID,1)
20     ContourMatrix_Area(cell_whiteID(i,2),cell_whiteID(i,1))=NaN;
21 end
22 ContourMatrix_Length=zeros(s_im(1),s_im(2));
23 for i=1:size(cell_whiteID,1)
24     ContourMatrix_Length(cell_whiteID(i,2),cell_whiteID(i,1))=NaN;
25 end
26 ContourMatrix_Circularity=zeros(s_im(1),s_im(2));
27 for i=1:size(cell_whiteID,1)
28     ContourMatrix_Circularity(cell_whiteID(i,2),cell_whiteID(i,1))=NaN;
29 end
30 [r,c]=find(w==1);
31 count=0;
32 allDetected = 0;
33 while sum(r)>0    [cell_whiteID,n_white,w,boundary]=RegionGrowing(w,r(1),c(1));
34     if n_white > 8
35         wingCells{end+1}= boundary;
36         count=count+1;
37         s_b=size(boundary,1);
38         all_l=[];
39         for i=1:s_b
40             l=sqrt(((boundary(i,1)-boundary(:,1)).^2)+(boundary(i,2)-boundary(:,2))
                   .^2);
41             all_l=[all_l;l];  %#ok
42         end
43         all_ll = [];
44         for i=1:size(boundary,1)-1
```

```
            ll=sqrt(((boundary(i,1)-boundary(i+1,1)).^2)+(boundary(i,2)-boundary(i+1,2)).^2);
            all_ll=[all_ll;ll];  %#ok
        end
        Perimeter = sum(all_ll);
        Rc = Perimeter/(2*pi);
        for i=1:size(cell_whiteID,1)
            if ~isempty(all_l)
                ContourMatrix_Area(cell_whiteID(i,2),cell_whiteID(i,1))=n_white;
                ContourMatrix_Length(cell_whiteID(i,2),cell_whiteID(i,1))=max(all_l);
                ContourMatrix_Circularity(cell_whiteID(i,2),cell_whiteID(i,1)) = n_white/(pi*(Rc^2));
            end
        end
        wingInfo(count,:) = [count...
        mean(boundary(:,1))...
        mean(boundary(:,2))...
        n_white...
        max(all_l)...
        n_white/(pi*(Rc^2))];
    end
    [r,c]=find(w==1);
    id_gray = w==0.1;
    w(id_gray) = 0;
end
id = ContourMatrix_Area == 0;
ContourMatrix_Area(id) = nan;
ContourMatrix_Area = ContourMatrix_Area(end:-1:1,:);
id= ContourMatrix_Length == 0;
ContourMatrix_Length(id) = nan;
ContourMatrix_Length = ContourMatrix_Length(end:-1:1,:);
id= ContourMatrix_Circularity == 0;
ContourMatrix_Circularity(id) = nan;
ContourMatrix_Circularity = ContourMatrix_Circularity(end:-1:1,:);
end
```

1.4 Code S4: Wing Geometry Extraction

Code S4, integrates Codes S1, S2 and S3 to extract geometric features of insect wing cells, and generates figures regarding the Cells area, length, and circularity.
Code Description:

- **Line 3:** To initiate wing image segmentation, the first function is *GetImage* (Code S1).
- **Lines 4-10:** These lines illustrate the *cellSegment* function. For this function, the *GetImage* function needs to be executed beforehand because it requires the binary image as input.
- **Lines 11-25:** In these lines, we demonstrate how to generate heatmaps of the wing cells' area, length, and circularity distribution.

Code S4: Integerating all functions to segment the wing

```
1 function wingGeometry
2 %% Import Image / Binarization
3 [imageBinaryImage,~,~,~]=GetImage;
4 %% Cell Segmentation
5 [ContourMatrix_Area,...
6     ContourMatrix_Length,...
7     ContourMatrix_Circularity,...
```

```
    wingOutline,...
    wingCells,...
    wingInfo] = cellSegment(imageBinaryImage);
% Figure 1: Area Distribution Filled Contour
fig1 = figure('Name','Wing Cells Area Distribution Filled Contour');
ax1 =gca(fig1);
contourf(ax1,ContourMatrix_Area,100,'linestyle','none')
axis(ax1,'equal')
% Figure 2: Wing Cells Length Distribution Filled Contour
fig2 = figure('Name','Wing Cells Length Distribution Filled Contour');
ax2 =gca(fig2);
contourf(ax2,ContourMatrix_Length,100,'linestyle','none')
axis(ax2,'equal')
% Figure 3: Wing Cells Circularity Distribution Filled Contour
fig3 = figure('Name','Wing Cells Circularity Distribution Filled Contour');
ax3 =gca(fig3);
contourf(ax3,ContourMatrix_Circularity,100,'linestyle','none')
axis(ax3,'equal')
end
```

2 Zenodo Repository

This article contains supplementary data on extracting geometric features from 389 insect wings. All data is documented in a Zenodo repository and can be accessed via the following link:
https://doi.org/10.5281/zenodo.10557201

3 Documented Files

In the repository, two folders are documented:

1. Dragonflies.zip
2. Damselflies.zip

The results for 102 damselflies and 287 dragonflies examined in this study are contained in the first and second folders, respectively. Each damselfly's and dragonfly's results are stored in separate folders named as follows:

Family-Genus-Species-Wing Position-(Wing Length)

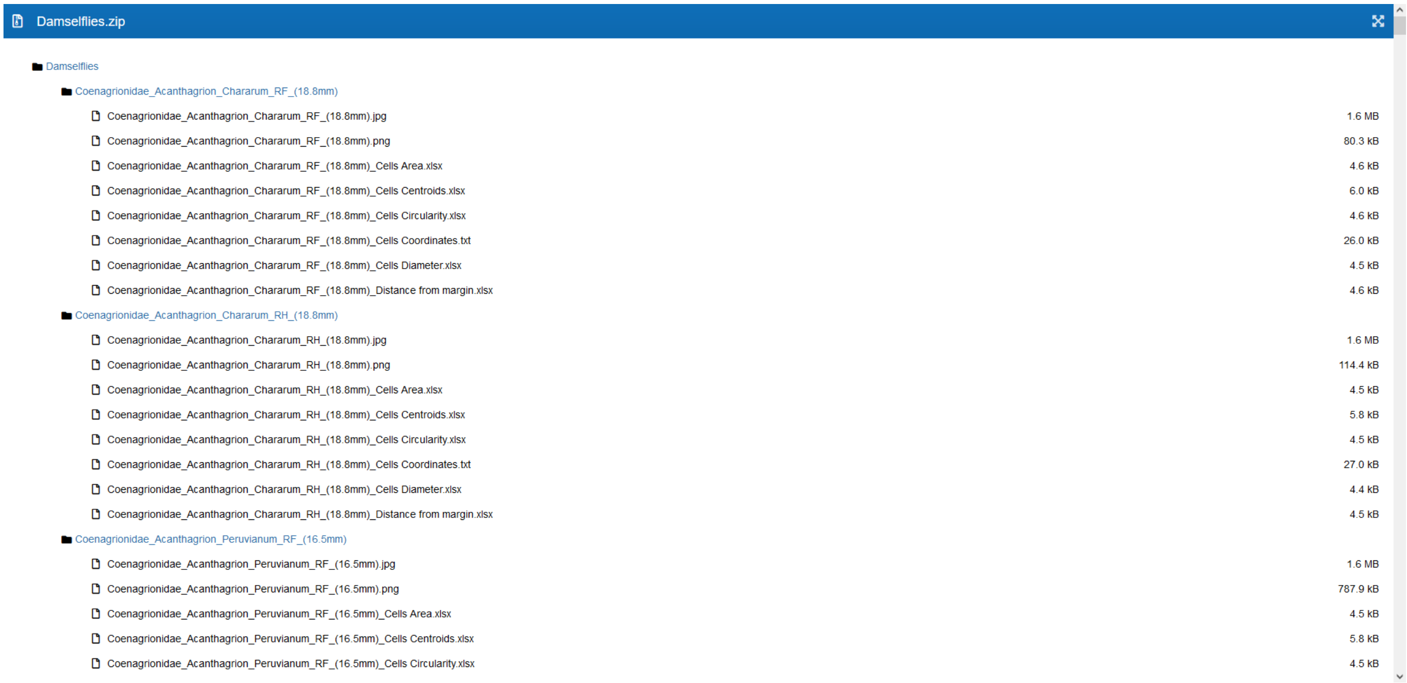

Figure S1: A screenshot of the Zenodo Repository containing the documents related to this paper

In each folder as depicted in Figure S1, there are two images related to the wing: one depicting the wing image and the other displaying the distribution contour, histogram, and box plots of cells' area, width, and circularity. Additionally, each folder contains five *.xlsx files storing information on Cells' Area, Centroids, Circularity, Diameter (Length), and cells' distance from margins. Furthermore, a *.txt file is included in each folder, providing the coordinates of cells and the outline of the wing.

4 WingSegment and WingGram

The software we employed for extracting the geometric features of insect wing cells, WingGram, can be accessed via the following link:
https://doi.org/10.5281/zenodo.7994584

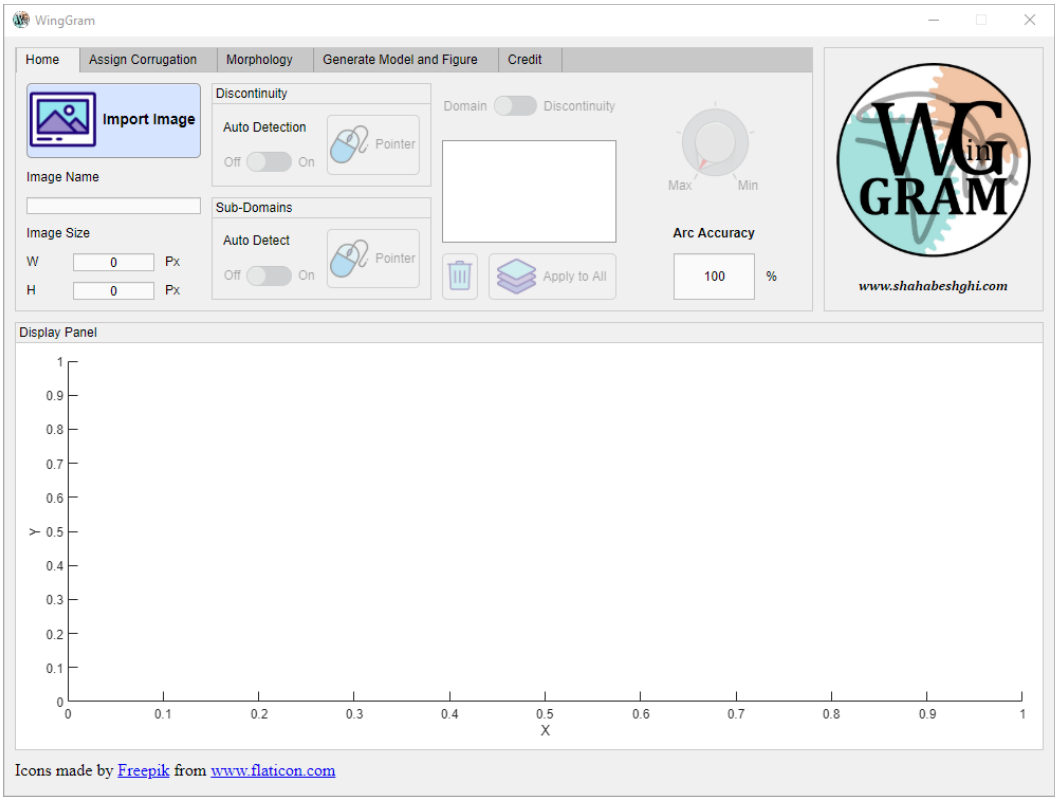

Figure S2: The main window of the WingGram software

WingSegment is currently under review and not yet published. It can be accessed by requesting the EXE file via the following link:
https://doi.org/10.5281/zenodo.8014588

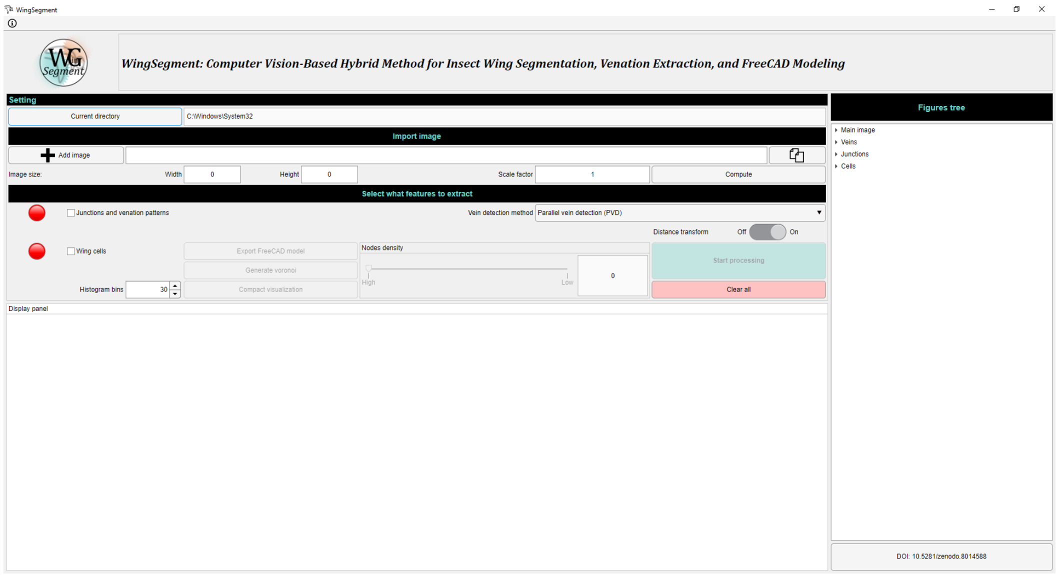

Figure S3: The main window of the WingSegment software

Both toolboxes are very user-friendly. Simply prepare the wing image and import it into the software using the embedded button. For more details about WingGram, you can refer to the following YouTube video:
https://www.youtube.com/watch?v=CMiDwAz-MWY&t=3s
